# Evaluating the Combination of Radioimmunotherapy and Immunotherapy in a Melanoma Mouse Model

**DOI:** 10.3390/ijms21030773

**Published:** 2020-01-24

**Authors:** R. Jiao, K.J.H. Allen, M.E. Malo, D. Rickles, E. Dadachova

**Affiliations:** 1 College of Pharmacy and Nutrition, University of Saskatchewan, Saskatoon, SK S7N 5E5, Canada; jiaorubin9712@hotmail.com (R.J.);; 2 RadImmune Therapeutics, Inc., Burlingame, 94010 CA, USA; drickles@radimmune.com

**Keywords:** DBA/2 mice, Cloudman S91 murine melanoma, C57Bl6 mice, radioimmunotherapy, anti-PD-1 immunotherapy

## Abstract

Immunotherapy has changed the oncology landscape during the last decade and become standard of care for several cancers. The combinations of immunotherapy with other treatment modalities are also being investigated. One of the challenges to investigate such combinations is to identify suitable mouse models for the pre-clinical experiments. In the past, we and other researchers showed that murine B16-F10 melanoma in C57Bl6 mice is refractory to treatment with immune checkpoint inhibitors. In this work we studied the suitability of an alternative syngeneic model, Cloudman S91 murine melanoma in DBA/2 mouse (DBA/2NCrl), to study the combination of immunotherapy targeting PD-1 and radioimmunotherapy targeting melanin. DBA/2 male and female mice were injected subcutaneously with 3–6 million Cloudman S91 cells. When the tumors reached ~150 mm^3^ volume, the animals were treated intraperitoneally with PBS (sham), h8C3 unlabeled (cold) antibody to melanin, immunotherapy with anti-PD-1 antibody, radioimmunotherapy with 213Bismuth (^213^Bi)-labeled h8C3 antibody, or several combinations of immunotherapy and radioimmunotherapy. Treatments with immunotherapy alone produced very modest effect on the tumor size, while combination therapy resulted in significant slowing down of the tumor growth, increased animal survival, and no decrease in animal body weight. We conclude that Cloudman S91 murine melanoma in DBA/2 mouse is a suitable model to evaluate combination of immunotherapy of melanoma with tangentially targeted treatments.

## 1. Introduction

Immunotherapy has changed the oncology landscape during the last decade and become standard of care for several cancers [1]. The combinations of immunotherapy with other treatment modalities are also being actively investigated [2]. One of the challenges to investigate such combinations is to identify suitable mouse models with intact immune systems for the pre-clinical experiments. It has been demonstrated that many syngeneic models are resistant to immunotherapy with checkpoint inhibitors [3]. 

Our laboratory is interested in developing therapies for metastatic melanoma. Metastatic melanoma was the first cancer for which immunotherapy with checkpoint inhibitors (ipilimumab as anti-CTLA-4 and pembrolizumab as anti-PD-1 inhibitors) was approved by US FDA. However, the results in clinical practice remain mixed, with only a minority of patients experiencing long-term progression free survival, at the expense of significant autoimmune toxicity [4]. Thus, clinical development of new therapeutics for melanoma to "wake up" the immune system to fight cancer is warranted, especially with agents possessing favorable toxicity profiles and mechanisms of action that differ from the currently approved checkpoint regulators. Radioimmunotherapy (RIT) targets radiation at the molecular level utilizing radiolabeled monoclonal antibodies (mAbs) that bind to over-expressed, or uniquely cancer specific, antigens located either on the cancer cell membrane, or in the extracellular space of the tumor microenvironment. These targeted radiopharmaceuticals can precisely deliver highly cytotoxic “internal radiation” to localized or systemic cancer deposits, while reducing potential side effects [5,6]. Effective RIT has the potential to open up new perspectives towards improving clinical outcomes in melanoma through modulation of antitumor immune response [7,8]. Several years ago we conducted a successful Phase 1 clinical trial in patients with metastatic melanoma using a murine antibody to melanin with an IgM isotype radiolabeled with a beta emitter 188Rhenium (^188^Re) [9]. This trial demonstrated safety and was indicative of the potential therapeutic efficacy of targeting melanin with radiolabeled antibodies. Since then, we developed a humanized IgG to melanin, which has proven to be efficacious in treatment of B16-F10 murine melanoma in female C57Bl6 mice when radiolabeled with an alpha emitter 213Bismuth (^213^Bi) [10]. It would be advantageous to evaluate a combination of RIT targeting melanin and immunotherapy with checkpoint inhibitors to achieve longer lasting responses to therapy. However, as B16-F10 melanoma in C57Bl6 mice is refractory to immunotherapy [3] we had to evaluate a different syngeneic model for such combination study. In this work we evaluated the suitability of Cloudman S91 murine melanoma in DBA/2 mice for the studies combining immunotherapy and RIT.

## 2. Results

**Figure 1:**
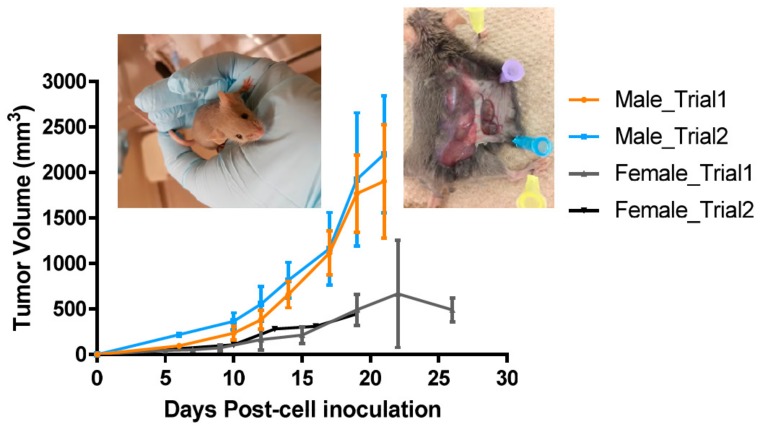
Cloudman S91 tumor growth in male and female DBA/2 brown mice. Five mice per group were injected subcutaneously with 3 or 6 million Cloudman S91 tumor cells into the right flank. Inserts contain the image of a brown mouse and a Cloudman S91 tumor after necropsy showing modest melanin concentration in the tumor.

Tumor development was more reliable in male DBA/2 mice. Figure 1 shows the results of the subcutaneous inoculation of Cloudman S91 tumor cells into the right flank of either male or female DBA/2 mice at the age of 6–9 weeks old. Tumor inoculation of both 3 and 6 million cells resulted in reliable tumor growth in male mice with tumors reaching 100–200 mm^3^ of the volume on Day 6 after inoculation. Only some female mice developed tumors that grew much slower, and several tumors stopped growing after Day 15. Based on these results, male mice were used in the follow-up combination therapy experiments.

**Figure 2:**
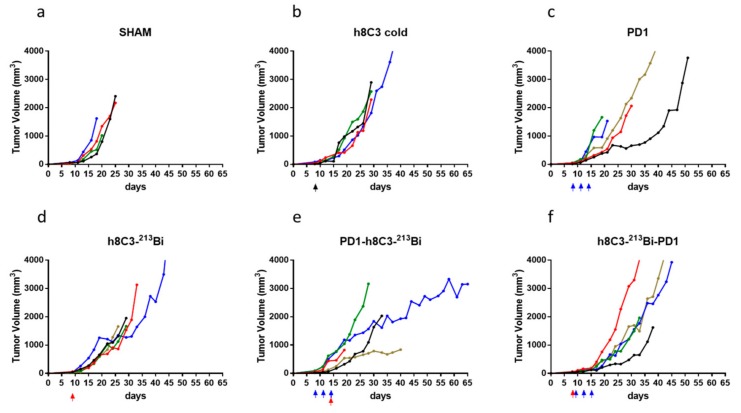
Combination therapy of Cloudman S91 tumor bearing male DBA/2 mice with anti-PD-1 mAb and one dose RIT with ^213^Bi-h8C3 mAb. The mice in groups of five were treated with: (**a**) sham injections with PBS; (**b**) a single dose of 80 µg unlabeled (cold) h8C3 on Day 8 after tumor cells inoculation; (**c**) three doses of 250 µg anti-PD-1 mAb on Days 8, 11, and 14; (**d**) one dose of 400 µCi ^213^Bi-h8C3 on Day 8; (**e**) three doses of anti-PD-1 mAb on Days 8, 11, and 14 and one dose of 400 µCi ^213^Bi-h8C3 on Day 14; (**f**) one dose of 400 µCi ^213^Bi-h8C3 on Day 8 and three doses of anti-PD-1 mAb on Days 9, 12, and 15. Each tumor volume curve represents a single animal. Black, blue, and red arrows indicate the injection of cold antibody, anti-PD1 antibody, and RIT respectively.

Combination of immunotherapy and RIT had more profound effect on the tumor growth than either therapy alone. Figure 2 displays the result of the experiment where immunotherapy was combined with one dose of RIT. Administration of a single dose of unlabeled melanin-targeting h8C3 mAb given on Day 9 after tumor inoculation did not have any effect on the tumor growth in comparison with mice given sham PBS injection (Figure 2a,b). Three doses of anti-PD1 mAb given on Days 8, 11, and 14 (Figure 2c) or a single RIT dose of ^213^Bi-8hC3 had modest effect (Figure 2d) on the tumors. Following the last anti-PD1 dose with a single RIT administration slowed down the tumor growth (Figure 2e), while reversing the order of RIT and immunotherapy seemed to make the combination less efficient (Figure 2f). 

**Figure 3:**
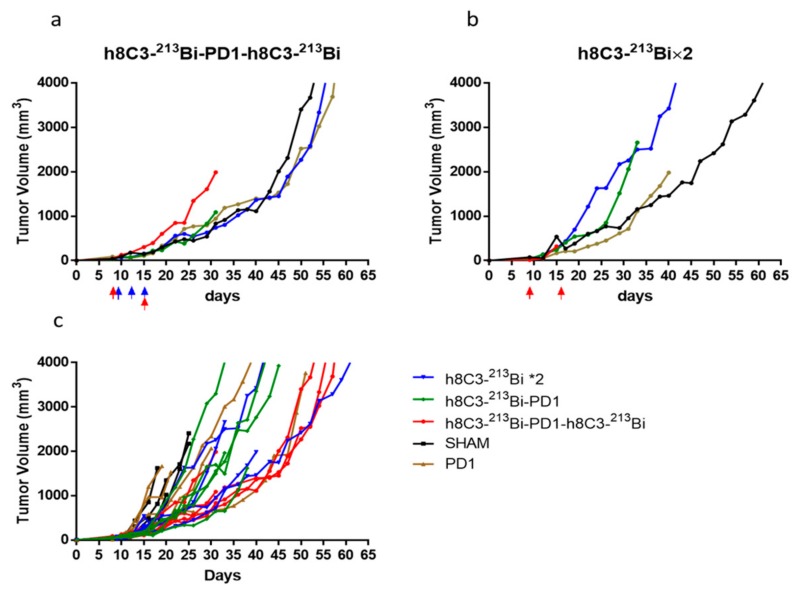
Combination therapy of Cloudman S91 tumor bearing male DBA/2 mice with anti-PD-1 mAb and two doses RIT with ^213^Bi-h8C3 mAb. The mice in groups of five were treated with: (**a**) two doses of 400 µCi ^213^Bi-h8C3 given on Days 8 and 15 and three doses of anti-PD-1 mAb given on Days 8, 11, and 15; (**b**) two doses of 400 µCi ^213^Bi-h8C3 given on Days 9 and 16; (**c**) combined plot for all treatment groups. Each tumor volume curve represents a single animal. Blue and red arrows indicate the injection of anti-PD1 antibody and RIT, respectively.

The next series of experiments combined two doses of RIT given on Days 8 and 15 with anti-PD-1 therapy “sandwiched” between RIT. This combination had a profound effect on slowing down the tumor growth (Figure 3a). The group given two doses of RIT alone had less pronounced effect on the tumor (Figure 3b). When the tumor growth curves from both experiments were compared to each other, two doses of RIT combined with anti-PD1 therapy had the most significant effect on the tumor out of all combinations (Figure 3c). When the time to achieve an average tumor size of 1500 mm^3^ was calculated, it was 26 days for cold h8C3, 28 days for anti-PD1 mAb alone, 31 days for two doses of RIT, and 43.5 days for the combination of two doses of RIT and anti-PD-1 mAb.

**Figure 4:**
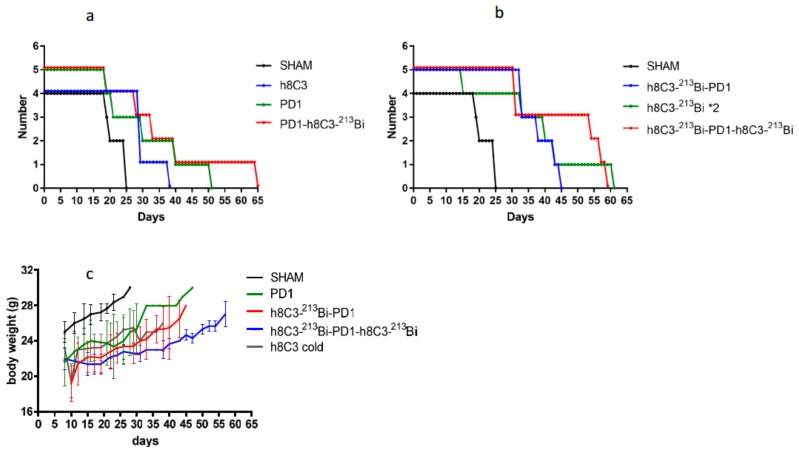
Survival and body weight of Cloudman S91 tumor bearing male DBA/2 mice treated with anti-PD-1 mAb and two RIT with ^213^Bi-h8C3 mAb: (**a**) sham, cold h8C3, three doses of anti-PD-1, and combination of three doses of anti-PD-1 mAb with one dose RIT with ^213^Bi-h8C3 mAb; (**b**) sham, combination of one dose RIT with ^213^Bi-h8C3 mAb followed with three doses of anti-PD-1 mAb, two doses of RIT with ^213^Bi-h8C3 mAb, and two doses of RIT with ^213^Bi-h8C3 mAb combined with three doses of anti-PD-1 mAb; (**c**) body weight of treated mice.

Combination therapy increased the survival and had minimal systemic toxicity. The combination of immunotherapy and RIT has an effect on the overall survival of the mice (Figure 4a, b), with mice given two doses of RIT and immunotherapy surviving the longest (Figure 4b). The combination therapy resulted in minimal systemic toxicity. After slight decrease in the body weight following RIT administration, mice in the RIT alone or combination groups recovered their body weight by approximately Day 22 and were gaining weight until the end of the observation period (Figure 4c).

## 3. Discussion

In pre-immunotherapy era, the majority of pre-clinical experiments on RIT of melanoma alone or in combination with other treatments such as chemotherapy were utilizing human melanoma cell lines inoculated into immunocompromised mice such as “nude” mice lacking T cells [11,12] or SCID (severe combined immunodeficiency mice) lacking both T and B cells [13]. The introduction of immunotherapy into the clinical care of melanoma patients in 2011 requires the use of immunocompetent animals in any experiments where immunotherapy is either used as a standard of care comparator or its combination with other therapeutic modalities are being investigated. Kuzu et al. provide a comprehensive review of mouse models in melanoma research [14]. In this work, we evaluated a suitability of Cloudman S91 murine melanoma in DBA/2 mice as a model for evaluating combination treatments of RIT with immune checkpoint inhibitors. We also took into consideration the cost effectiveness of this model, as DBA/2 is the oldest inbred mouse strain having been bred for more than 100 years.

Cloudman S91 is one of the several melanized murine cell lines, with others being B16, B16-F1, B16-F10, and Harding-Passey cells [15,16,17]. The degree of melanization of these cell lines in vitro depends on the presence of several stimulants of melanin synthesis such as alpha-melanocyte stimulating hormone (α-MSH), retinoic acid, etc., [15,16]. To the best of our knowledge, there has not been a quantitative comparison of melanin amount produced by these cell lines when used for tumor initiation in mice. In our visual observations, the amount of melanin produced by B16-F10 *in vivo* is higher than that produced by Cloudman S91. There has been some controversy in regard to PD1 expression by the wild type B16-F10 cell line—while Kleffel et al. demonstrated some expression; their therapy results with anti-PD1 antibodies in C57Bl6 mice were disappointing [18]. Currently wild type B16-F10 is considered refractory to anti-PD1 and anti-CTLA4 checkpoint inhibitors [3]. Cloudman S91 expresses PD1 [19] and is responsive to immunotherapy with checkpoint inhibitors [3]. It has been used for evaluating the combination of anti-PD1 with beta-alethine [20] and of anti-PD-L1 with anti-VEGF therapies [21].

The Cloudman S91 grew reliably and aggressively in male DBA/2 mice but very slowly in female pointing to possible hormonal dependence of the tumor growth. In this regard, such profound difference in tumor aggressiveness between male and female mice resembles the mortality of men from metastatic melanoma, which is almost double that of women (American Cancer Society data). This observation also emphasizes the importance to consider sex when developing animal models for cancer treatment. 

While the effects of anti-PD1 therapy on Cloudman S91 melanoma in DBA/2 mice have been reported [3], to the best of our knowledge, this is the first study in which RIT targeting melanin was evaluated in this model. Cloudman S91 tumors contain much less melanin that is a target for h8C3 mAb than B16-F10 melanoma tumors (insert in Figure 1), which explains why the effect of ^213^Bi-h8C3 RIT on Cloudman S91 tumors was less impressive than on B16-F10 tumors in C57Bl6 mice [10]. However, our previous attempt to combine RIT targeting melanin with checkpoint inhibitors in B16-F10/C57Bl6 model proved complete inefficiency of checkpoint inhibitors in that model [22], confirming the data reported in [3]. In contrast, the combination of two doses of RIT with anti-PD1 therapy effectively slowed down the Cloudman S91 tumor growth by 1.5 times and increased the animal survival with no appreciable systemic toxicity.

We conclude that Cloudman S91 murine melanoma in DBA/2 male mice is suitable for evaluating the combination of immunotherapy with targeted radionuclide therapies such as RIT. The future studies in this model will include the evaluation of the complementary effects of RIT on the priming and effector phases of antitumor T cell immunity. Understanding the ability of an intervention with RIT to induce a *de novo* antitumor immune response will shed significant light on how to tilt the balance from an immune-suppressive to an immune-active environment for effective anti-melanoma therapy.

## 4. Materials and Methods

Antibodies, reagents and radionuclides. Aragen Bioscience (Morgan Hill, CA, USA) manufactured the humanized 8C3 mAb (h8C3). A ^213^Bi/^225^Ac radionuclide generator was produced via ^225^Ac purchased from Oak Ridge National Laboratory (Oak Ridge, TN, USA). Macrocyclics (Dallas, TX, USA) synthesized the bifunctional chelating agent (BCA) N-[2-amino-3-(p-isothiocyanatophenyl)propy1]-trans-cyclohexane-1,2-diamine-N,N’,N’’,N’’’,N’’’’-pentaacetic acid (CHXA"). Rat IgG2a to mouse PD-1 (Programmed death-1) also known as CD279 was acquired from Bio X Cell (West Lebanon, NH, USA).

Conjugation of BCA CHXA’’ to h8C3 antibody and radiolabeling with ^213^Bi. The conjugation of CHXA” to h8C3 was performed as in [23] with a minor alteration. A 10-fold molar excess of CHXA’’ was used in place of a 5-fold excess. Radiolabeling with **^213^Bi** was performed analogously to that described in [10].

Murine Cloudman S91 melanoma model. All animal studies were approved by the Animal Research Ethics Board of the University of Saskatchewan (Animal use protocol #20170006, approved on 28 February 2019). Six-eight weeks old male and female DBA/2 mouse (DBA/2NCrl) were purchased from Charles River. Mice were injected subcutaneously into the right flank with 3–6 million Cloudman S91 cells and the tumors were measured every three days.

Combination treatment anti-PD-1 immunotherapy and RIT. The male DBA/2 mice bearing subcutaneous Cloudman S91 with approximately 150 mm^3^ were randomized into the groups of five and treated with either: sham injections with PBS; a single dose of 80 µg unlabeled (cold) h8C3 on Day 9 after tumor cells inoculation; three doses of 250 µg anti-PD-1 mAb on Days 8, 11, and 14 as in [24]; one dose of 400 µCi ^213^Bi-h8C3 on Day 8; three doses of anti-PD-1 mAb on Days 8, 11, and 14 and one dose of 400 µCi ^213^Bi-h8C3 on Day 14; one dose of 400 µCi ^213^Bi-h8C3 on Day 8 and three doses of anti-PD-1 mAb on Days 9, 12, and 15. In another experiment the tumor bearing mice were treated with either two doses of 400 µCi ^213^Bi-h8C3 given on Days 9 and 16 or two doses of 400 µCi ^213^Bi-h8C3 given on Days 8 and 15 and three doses of anti-PD-1 mAb given on Days 9, 12, and 15. Any mouse which tumor became necrotic or started to interfere with its well-being was humanely sacrificed. The body weight of the mice was measured every three days and they were also assessed visually using body conditioning score.
